# Frequency and Genetic Characterization of *Echinococcus granulosus* in Paraffin‐Embedded Human Tissue Samples From the Northwest of Iran, West Azarbayjan

**DOI:** 10.1155/japr/6008555

**Published:** 2026-06-20

**Authors:** Mahsa Boustani, Shahram Khademvatan, Elham Yousefi, Farahnaz Noroozinia, Saber Gholizadeh

**Affiliations:** ^1^ Cellular and Molecular Research Center, Cellular and Molecular Medicine Institute, and Department of Medical Parasitology and Mycology, Urmia University of Medical Sciences, Urmia, Iran, umsu.ac.ir; ^2^ Department of Veterinary Biosciences, Melbourne Veterinary School, Faculty of Science, The University of Melbourne, Parkville, Victoria, Australia, unimelb.edu.au; ^3^ Department of Pathology, School of Medicine, Urmia University of Medical Sciences, Urmia, Iran, umsu.ac.ir; ^4^ Department of Medical Entomology, School of Health Social Determinants of Health Research Center, Clinical Research Institute, Urmia University of Medical Sciences, Urmia, Iran, umsu.ac.ir

**Keywords:** *Echinococcus granulosus*, formalin-fixed paraffin-embedded, frequency, genotypes, human

## Abstract

**Objectives:**

Cystic echinococcosis (CE), caused by the *Echinococcus granulosus* larval stage, poses health problems in the world, including Iran. This study is aimed at investigating the epidemiological and molecular characterization of *E. granulosus* collected from CE samples in Urmia, the northwest of Iran.

**Methods:**

In this cross‐sectional study, the demographic information of 295 hydatid cyst patients who underwent surgery between 2010 and 2021 was recorded and analyzed statistically. Due to time and financial constraints, a total of 74 samples were evaluated. DNA of FFPE (formalin‐fixed paraffin‐embedded) hydatid cyst samples was extracted, and PCR was performed using mitochondrial genes *cox1* and *nad1*. PCR products were electrophoresed and sequenced, and sequence analysis was performed using BioEdit and BLAST software.

**Results:**

Among 295 cases studied, 173 (58.64%) were female and 122 (41.36%) male, respectively, and the CE frequency was significantly higher in patients aged 20–30 (*n* = 24/295; 8.1%), villagers (*n* = 70/295; 23.7%), and low educated cases (*n* = 82/295; 27.8%). The most group affected by CE was the housewives (*n* = 33/295; 11.2%), followed by the illiterate people (*n* = 82/295; 27.8%) and the farmers (*n* = 17/295; 5.8%). The liver (*n* = 52/295; 17.6%) and the lung (*n* = 40/295; 13.6%) were the most common sites for cyst formation, followed by the abdomen (*n* = 9/295; 3.1%), kidney (*n* = 4/295; 1.4%), thorax (*n* = 2/295; 0.7%), spleen (*n* = 2/295; 0.7%), and the pancreas (*n* = 3/295; 1.0%). DNAs from all 74 paraffinized hydatid cyst samples were extracted successfully. Of the whole FFPE samples amplified by PCR assay using nad1 and cox1 genes, only 27 and 25 FFPE samples were sequenced, respectively. The 9.1% (*n* = 27) for cox1 and the 8.4% (*n* = 25) for nad1 were sequenced. All samples′ analyses resulted G1. In addition, analysis of cox1 and nad1 genes did not identify any haplotypic variation. DNAs from all 74 paraffinized hydatid cyst samples were extracted successfully. Of the whole FFPE samples amplified by PCR assay using *nad1* and *cox1* genes, only 27 and 25 FFPE samples were sequenced, respectively. The 9.1% (*n* = 27) for *cox1* and the 8.4% (*n* = 25) for *nad1* were sequenced. All samples analyzed resulted G1. In addition, analysis of *cox1* and nad1 genes did not identify any haplotypic variation.

**Conclusions:**

Molecular findings identified the G1 genotype as the predominant genotype involved in *E. granulosus* transmission in the northwest region of Iran.

## 1. Introduction

Echinococcosis is a serious, occasionally fatal, pathogenic condition. This globally significant zoonotic parasitic disease is caused by infection with the larval stages of the *Echinococcus granulosus sensu lato* (*s.l.*), a tapeworm belonging to the Cestoda class and the Taeniidae family. *E. granulosus* and *E. multilocularis* are two major species of *Echinoccoccus* of medical and public health importance and cause cystic echinococcosis (CE) and alveolar echinococcosis (AE) [1]. CE is more common in areas where sheep and goats breed because animals, as reservoirs of parasites or hosts, mediate in the evolution of the disease, and sometimes, human enters this evolutionary stage by chance [2]. In humans, the life span of hydatid cysts of *E. granulosus* can vary from 16 to 53 years, respectively [3, 4]. The symptoms vary depending on the size of the cyst, organ involved, association of this organ with other organs, integrity of the cyst wall, and factors such as genotypes [5].

CE has a global distribution with worldwide annual incidence rate of 1–200 per 100,000 [6] and is a major endemic health problem in certain areas of the world [1]. It is also endemic in various cattle‐ and sheep‐raising geographic regions, such as Mediterranean countries, the Middle East, Eastern Europe, and South America [7]. In Iran, CE is being actively transmitted and the annual surgical incidence rate is estimated to be 0.80–1.73 per 100,000 [8], imposing a significant financial and medical burden in the country [8, 9]. Surveys conducted in Iran have reported varying seroprevalence rates of CE, 5.9% in the center (Shahriar city), 13.8% in the southwest (Khuzestan Province), 4.8% in the west (Chaharmahal and Bakhtiari Province), and 7% in the east (Mashhad city) [10].

Humans are considered dead‐end intermediate hosts for CE. The clinical symptoms of the disease vary from asymptomatic to symptomatic, that is, development of hydatid cyst in the lung, liver, spleen, and other body organs. *E. granulosus* has special forms of phenotypes that are distinguishable based on their morphological and biochemical characteristics, but such strategies are unable to accurately identify the diversity of this species and its interspecies variation. Moreover, a high degree of genetic diversity is observed in this parasite [1]. By applying modern and highly sensitive molecular methods, researchers could discriminate a complex intraspecific strain of *E. granulosus* and displayed that different group of genetic alternatives comprised the genotype of *E. granulosus*. Sequence comparison of mitochondrial DNA (mtDNA) is a molecular technique that simply identifies genetic diversity and DNA polymorphism using appropriate genetic markers, for example, mtDNA cytochrome c oxidase (*cox1*) and Nicotinamide adenine dinucleotide *(nad)* genes, in both intermediate and definitive hosts [5]. This method contributes to the clarification of epidemiological situations and control of CE in a particular area because genetic diversity affects the epidemiology and pathogenicity of the disease [11]. To categorize *E. granulosus* genotypes into distinct genotypes, analysis of mitochondrial and nuclear genomes is needed.

Based on phenotypic features and gene sequence analysis, to date, different genotypes (G1‐G10) have been identified for *E. granulosus*. Except for G10, all the genotypes have been reported from humans. Also G9 is no longer recognized as a distinct genotype; it is probably a microvariant of G7 and G2 is considered a microvariant of G3. G1, so‐called sheep genotype, is the most prevalent human genotype reported globally [12].

This genetic characterization and the extensive different variants are remarkable for better understanding the life cycle pattern and other biological characteristics of *E. granulosus*, such as sensitivity to chemotherapy and pathologic patterns. In Iran, genotypes G1, G3, and G6 are common among livestock, G1and G3 are mostly widespread among dogs, and G1 and G6 are prevalent in humans [13]. In the west of the country, varied genotypes of *E. granulosus* have been detected in human, as well as in cattle, sheep, camel, and buffalo isolates using molecular methods; however, the parasite in paraffin‐embedded tissue of hydatid cysts isolates from human have not formerly been distinguished in the northwest of Iran. Therefore, the sources of infection in humans and role of intermediate host reservoirs remain to be illuminated. Identification of varying genotypes of *E. granulosus* could help the disease control programs by improving the understanding of transmission patterns, host specificity, and sources of infection, particularly in endemic areas. In a comprehensive molecular study investigating the occurrence of *E. granulosus* in formalin‐fixed paraffin‐embedded (FFPE) tissue specimens in Turkey, only 29 (41.6%) out of 70 total blocks could be genotyped [14]. In another survey of human CE, 30 samples of FFPE tissue were studied, but the method used was the restriction enzyme analysis of ITS1 region, which cannot accurately differentiate genotypes of *E. granulosus* [15].

Due to the fact that in northwest Iran, limited research work have hitherto been conducted on the FFPE tissues of human CE to study *E. granulosus* genotypes, identify the dominant genotype and determine the relationship of different genotypes with the organ involved in suspicious cases, this study was undertaken to investigate the frequency of CE and determine the genotype of the parasite in the above‐mentioned FFPE samples archived in the pathology department of Imam Khomeini Hospital, Urmia, the northwest of Iran.

## 2. Materials and Methods

### 2.1. Sample Collection

The present study was conducted in Urmia city, the northwest of Iran, between 2010 and 2021. Urmia is located in West Azerbaijan Province in northwest Iran. The geographical coordinates of this city are latitude 37.5498° N, and longitude 45.0786° E, with a Mediterranean climate. FFPE tissues were collected from 295 cases with histologically confirmed CE. Patients′ data, including gender, age, occupation, education, location (all parts of West Azerbayjan Province), and affected organs, were recorded. Overall, 74 out of 295 tissue samples were selected for molecular tests. All the patients were detected to be infected with CE by a pathologist. For each patient, 5–6 sections of 6 *μ*m thickness from tissue blocks were prepared, and excess paraffin was removed. Following placing in 1.5 mL microtubes, tissue sections were deparaffinized with xylene (1 mL) at 37°C for 10 min. Afterwards, the samples were centrifuged at 2060 × g at room temperature for 5 min, and the supernatant was eliminated; this procedure was repeated again. After deparaffinization, rehydration was performed in 100%, 90%, 80%, and 70% ethanol according to Schneider et al. [16]. In the end, after the removal of 70% ethanol, for DNA extraction, the tissue lysis solution was added.

### 2.2. DNA Extraction

Samples were washed twice with phosphate‐buffered saline for the removal of ethanol. To obtain parasite genome, we performed various extraction methods and finally applied Schneider et al.′s [16] technique with some modifications. A commercially available DNA extraction kit (SinaClon DNA Extraction Kit, Tehran, Iran) based on a simple salting‐out procedure was used. The protocol included a modified proteinase K step with prolonged digestion (up to 48 h) and repeated addition of proteinase K aliquots until lysis was complete [16]. The final proteinase K concentration was approximately 500 *μ*g/mL, and incubation was done at 56°C with agitation. Extracted DNAs were preserved at −20°C for further use.

### 2.3. PCR

PCR reaction was performed using two forward and reverse primers: JB3 (5 ^′^‐TTTTTTGGGCATCCTGAGGTTTAT‐3 ^′^) and JB4.5 (5 ^′^‐TAAAGAAAGAACATAATG AAAATG‐3 ^′^) and MS1 (5 ^′^‐CGTAGGTATGTTGGTTTGTTTGGT‐3 ^′^) and MS2 (5 ^′^‐CCATAATCAAATGGCGTACGAT‐3 ^′^) for *cox1* and *nad1*, respectively [17]. PCR was also utilized to amplify the fragments of 400 and 450 bp for mitochondrial genes, *cox1* and *nad1*, respectively. PCR amplification was accomplished in a reaction volume of 20 *μ*L comprising 0.025 U/*μ*L of Taq DNA polymerase, 75 mM of Tris‐HCl (pH 8.5), 20 mM of (NH_4_)SO_4_, 0.2 mM of deoxyribonucleosides, 5 *μ*L of template DNA, and 1 *μ*L of each forward and reverse primer (at 0.2 *μ*m concentration). The amplification conditions for both genes were adjusted as follows: initial denaturation at 95°C for 10 min, and then by 45 cycles (denaturation), annealing at 50°C and 56°C for the *cox1* and *nad1*, respectively, for 1 min, with a final extension step at 72°C for 10 min. PCR products were analyzed by 2.5% agarose gel containing safe stain as a nucleic acid stain, and the results of analysis were evaluated using a transilluminator device.

### 2.4. DNA Sequencing and Phylogenetic Analysis

Both *cox1* and *nad1* genes were bidirectionally sequenced from the selected PCR amplicons by Bioneer (Daejeon, Korea) briefly, PCR products were purified from agarose gel using the MinElute gel extraction kit (QIAGEN Ltd., Hilden, Germany) to enhance read quality, and gel‐extracted DNA, and analyzed by Chromas software Version 1.0.0.1. The BioEdit software (Version 7.2) was used to compare the edited sequences. Nucleotide queries of the present study were submitted to the GenBank database, and coding sequences were aligned by multiple sequence alignments using hierarchical cluster analysis, genotype was also determined. Based on the DNA sequences of the *nad1* and *cox1* genes, haplotype diversity was constructed using the DNASP V6 software. A phylogenetic tree was created with the aid of maximum likelihood method based on the Tamura–Nei model with 500 bootstrap replicates (MEGA10).

### 2.5. Data Analysis

Normality of data distribution was assessed using the Shapiro–Wilk test. All the data were statistically analyzed with Mann–Whitney and Kruskal–Wallis *H* tests using SPSS Version 16. The probability level of 0.05 was accepted as statistically significant. Moreover, nonparametric Fisher′s exact and chi‐square tests were used to measure the correlation between the parameters.

## 3. Result

### 3.1. Demographic Information

**Figure 1:**
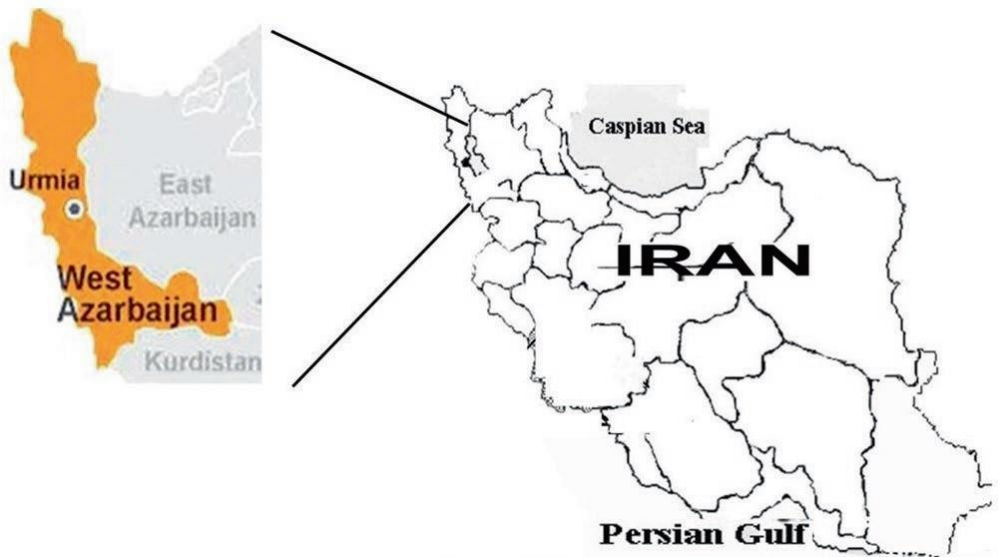
Geographical regions of the northwest of Iran (West Azarbaijan Province, Urmia city) where cystic echinococcosis samples were collected (orange regions).

In the present descriptive cross‐sectional study, 295 samples of archived paraffinized hydatid cysts were examined for the demographic information. The samples were collected from a hospital in the northwest of Iran between 2010 and 2021 (Figure 1). Table 1 represents the demographic data of these CE patients.

**Table 1:** Demographic variables and risk factors associated with cystic echinococcosis in patients who underwent surgery between 2010 and 2021 in northwest Iran.

Variable		*N*	(%)	*p*
Gender				
Female		173	(58.64)	0.017
Male		122	(41.36)	
Age (y)				
< 10		22	(7.5)	0.003
11–20		30	(10.2)	
21–30		71	(24.1)	
31–40		60	(20.3)	
41–50		45	(15.3)	
51–60		38	(12.9)	
> 61		29	(9.8)	
Residency				
Rural		207	(70.17)	0.012
Urban		88	(29.83)	
Education level				
Never went to school and literacy class		243	(82.4)	0.007
Diploma		30	(10.2)	
Associate		5	(1.7)	
Bachelor		14	(4.7)	
Upper		3	(1)	
Occupation				
Employee		7	(2.4)	0.019
Farmer		51	(17.3)	
Animal husbandry		24	(8.1)	
Housewife		98	(33.2)	
Student		31	(10.5)	
Unemployed		34	(11.5)	
Other		50	(16.9)	
Risk factors				
Close contact with dog	Yes	210	71.19	< 0.001
	No	85	28.81	
Sheep slaughter	Yes	237	80.34	< 0.001
	No	58	19.66	
Eating unwasahed vegetables	Yes	141	47.8	0.212∗
	No	154	52.2	
Prior knowledge about disease	Yes	140	47.46	0.379∗
	No	155	52.54	
Total		295	100	

*Note:* ∗, Nonsignificant.

The highest and lowest frequency of CE were observed in the age range of 21–30 years (24.1%) and less than 10 years (7.5%), respectively. A significant association was observed between CE infection and gender (*p* = 0.017), residency (*p* = 0.012), education level (*p* = 0.007), occupation (*p* = 0.019), and risk factors including close contact with dogs and sheep slaughtering (*p* < 0.001). In both sex groups, the liver was found to be the most common infected organ, followed by the lungs and kidneys, and the spleen was the least involved organ. Also, there was no significant relationship between gender and the involved organ (Table 2).

**Table 2:** Anatomical sites of hydatid cysts (*n* = 295).

Affected organs	Female %	Male %	Total (%)	
Lung	78 (45.08)	50 (40.98)	128 (43.03)	
Liver	88 (50.86)	59 (48. 36)	147 (49.61)	
Spleen	1 (0.57)	1 (0.81)	2 (0.69)	Pgender − organ = 0.325
Kidney	1 (0.57)	3 (2.45)	4 (1.51)	
Pancreas	1 (0.57)	2 (1.63)	3 (1.11)	
Abdomen	4 (2.31)	5 (4.09)	9 (3.20)	
Thorax	—	2 (1.63)	2 (0.85)	
Total	173	122	295 (100)	

**Figure 2:**
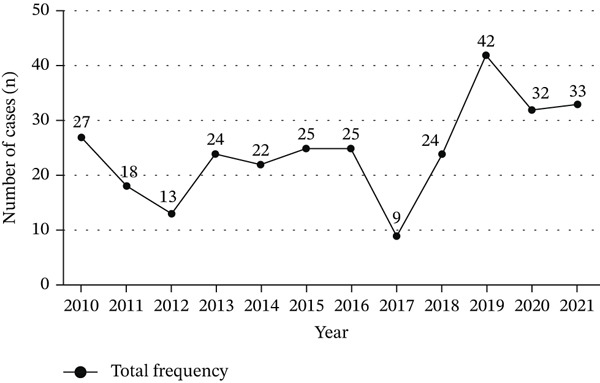
Annual distribution of cystic echinococcosis cases recorded between 2010 and 2021 in northwest Iran.

The highest and lowest percentages of case of infections detected during the above‐mentioned periods were 14.24% in 2019 and 4.41% in 2012, respectively (Figure 2). The results showed that close contact with dog (71.19%; *p* < 0.05) and history of sheep slaughtering (80.34%; *p* < 0.05) as risk factors were statistically significant (Table 1).

### 3.2. Molecular Analysis

All DNA isolates extracted from 74 FFPE hydatid cysts were subjected to molecular analysis targeting the *cox1* and nad1 genomic fragments.

The *cox1* and *nad1* amplification was successfully accomplished on 74 out of 295 samples. However, only 27 samples (for the *cox1* gene) and 25 samples (for *nad1* gene) were properly sequenced. The final edited sequence of *cox1* fragment was 450 bp, whereas that of *nad1* was 400 bp. Overall, all the samples of the positive isolates belonged to the G1 genotype. Sequence analysis of the PCR product was performed using the NCBI BLAST (Table 3).

**Table 3:** Accession numbers and infected organs in sequenced samples for *nad1* and *cox1* genes.

No	Gender	Age	Affected organ	Accession numbers	
				*nad1*	*cox1*
1	M	26	Right lobe of the liver	MZ267694	MZ093082
2	M	33	Right lobe of the liver	MZ267695	MZ093083
3	F	61	Right lung	MZ267696	MZ093084
4	M	65	Left lobe of the liver	MZ267697	MZ093085
5	F	50	Left lobe of the liver	MZ267698	MZ093086
6	F	44	Right lobe of the liver	MZ267699	MZ093087
7	F	33	Left lobe of the liver	MZ267700	MZ093088
8	M	75	Right lung	MZ267701	MZ093089
9	F	46	Right lobe of the liver	MZ267702	MZ093090
10	F	28	Left lobe of the liver	MZ267703	MZ093091
11	M	36	Right lung	MZ267704	MZ093092
12	F	39	Left lobe of the liver	MZ267705	MZ093093
13	M	59	Right lobe of the liver	MZ267706	MZ093094
14	M	65	Upper lobe of the liver	MZ267707	MZ093095
15	F	62	Left lobe of the liver	MZ267708	MZ093096
16	M	39	Lower lobe of the liver	MZ267709	MZ093097
17	F	41	Kidney	MZ267710	MZ093098
18	M	25	Right lung	MZ267711	MZ093099
19	M	26	Lower lobe of the liver	MZ267712	MZ093100
20	F	56	Right lobe of the liver	MZ267713	MZ093101
21	F	66	Pancreas	MZ267714	MZ093102
22	M	38	Upper lobe of the lung	MZ267715	MZ093103
23	F	37	Right lobe of the liver	MZ267716	MZ093104
24	F	71	Upper lobe of the liver	MZ267717	MZ093105
25	M	76	Right lobe of the liver	MZ267718	MZ093106
26	M	63	Upper lobe of the liver	—	MZ093107
27	F	19	Left lobe of the liver	—	MZ093108

Abbreviations: F, female; M, male.

**Figure 3:**
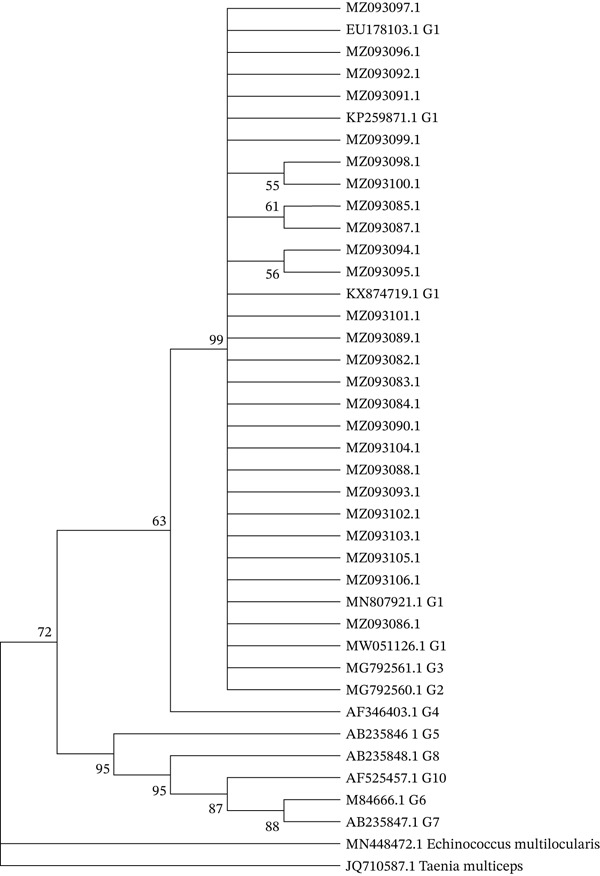
A distance‐based maximum likelihood cladistic tree of *Echinococcus granulosus* G1 genotype based on the *cox1* gene only bootstra *p* values higher than 70% are indicated on each branch. Taenia multiceps (Accession No: JQ710587) *Echinococcus multilocularis* (*MN448472.1*) was used as the out‐group.

**Figure 4:**
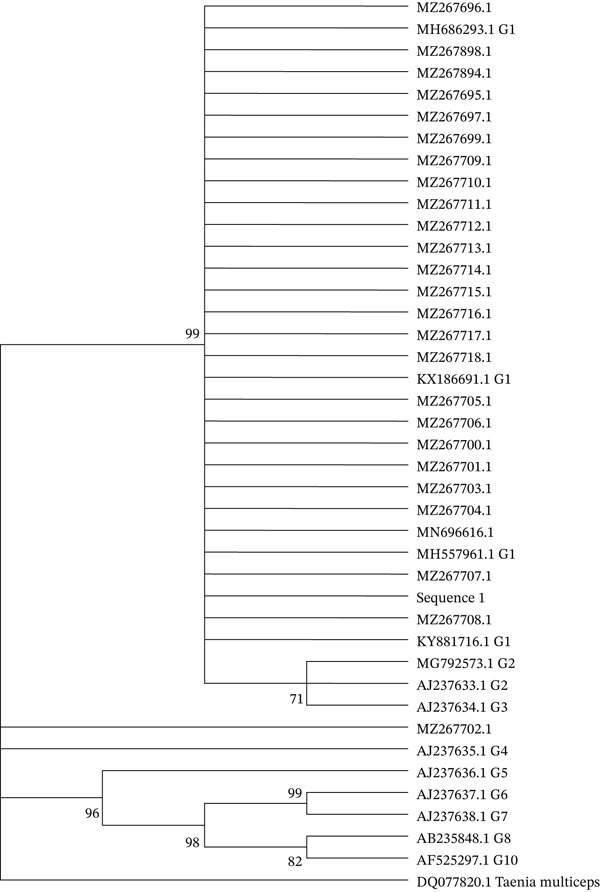
A distance‐based maximum likelihood cladistic tree of *Echinococcus granulosus* G1 genotype based on the *nad1* gene. Only bootstra*p* values higher than 70% are indicated on each branch. The collected location characterized genotype (G1) and registered accession numbers marked by geometric shapes. Taenia multiceps (Accession No DQ077820.1) was considered an out‐group branch.

Analysis of *cox1* and *nad1* gene sequence of varied samples did not identify any haplotypic variation in the G1 genotypes. The sequences obtained from *cox1* and *nad1* genes were submitted to the GenBank with the accession numbers MZ093082‐MZ093108 and MZ267694‐MZ267718, respectively (Figures 3 and 4).

## 4. Discussion

For many years, CE has been recognized as an important health problem worldwide, particularly in several countries such as the Middle East, including Turkey, Iraq, Pakistan, and Iran [8, 18, 19].

Findings from the present study offer the first insight into *E. granulosus s.l.* genetic diversity in FFPE samples in Urmia, the northwest of Iran. The majority of people, who live in Urmia, due to their work in agriculture and animal husbandry, are exposed to soil‐transmitted diseases, especially CE and other parasitic infections, like in several other countries, as reported by other studies [20–22]. Moreover, Urmia has a large number of stray and shepherd dogs that are in close contact with humans and other animals, including sheep and goats [23].

In Iran, as in other parts of the world, there are various (cultural, socioeconomical, educational, agricultural, and environmental) factors contributing to the transmission of infection [24]. Other factors assist in the domestic cycles of transmission include insufficient knowledge and education about the *E. granulosus s.l.* life cycle and the absence of legislation for meat hygiene and inspection during abattoirs, as well as offal disposal at local abattoirs [10, 22, 25].

In the present study, genotyping of human CE isolates provided useful epidemiological information regarding transmission patterns in the study area. The strain variation in parasite is also thought to influence life‐cycle patterns, host specificity, transmission dynamics, development rate, antigenicity, and sensitivity to chemotherapeutic drugs. Thus, identification of genotypes could help develop and design vaccines, as well as improve diagnostic and therapeutic methods [3].

In our study, patients aged 20–30 years were identified as the main sufferers of CE, but in other studies conducted in Italy and Turkey, the disease was more common among middle‐aged and elderly patients [14, 26]. As the most active age groups of the society are people in the age range of 20–30 years, CE could have destructive economic damages. This finding may be associated with greater occupational and environmental exposure of young adults to livestock, dogs, and contaminated environments in rural areas.

In the adult group of our study, the frequency of CE in women particularly housewives, was higher than men, which confirm previous studies in Iran. This may be related to closer contact of rural women with contaminated vegetables, domestic animals, and food preparation activities. Similar results were also disclosed in Khademvatan et al.′s [27] study in Iran in which women (4.5%) were more affected than men. Conchedda et al.′s [26] investigation in Italy reported a reverse result; the ration of male to female patients was 1.36%.

In some areas of Iran, women, particularly those living in rural areas, are accustomed to picking mountain vegetables grown near springs and waterways. It has sometimes been observed that these vegetables are contaminated with parasite eggs, and without being washed or disinfected, they are eaten, and this causes disease in these women.

Evaluation of organ distribution of cysts suggested that varying organs were involved in CE; however, the liver was the most organ affected. This observation was in agreement with the results of other studies [24, 28], which reported the liver as the most common organ for cyst formation. Although the liver and lungs are the most frequent involved organs (49.61%, 43.03%), respectively, hydatid cyst is localized in rare sites including the abdomen (3.20), spleen (0.69), kidney (1.51), and the pancreas (1.11). In a survey conducted in Tehran Province (the center of Iran), Sadjjadi et al. [29] implied a link between genotypes and involved organ and indicated that G6, compared to G1, has a higher affinity for the human brain. They found that all eight samples of human FFPE have the G6 strain, and, therefore, expressed the possibility of tropism of genotypes to specific organs. However, this finding is more dependent on the genotype being the most common in intermediate and definitive hosts in that particular area. In another investigation in Isfahan Province (in the center of Iran), Jafari et al. [30] used *cox1* and *nad1* genes and reviewed 50 surgical specimens of hydatid cyst. They introduced G1, G3, and G6 as the most common genotypes, respectively; however, all six samples identified in the G6 strain were related to the liver, and no brain samples were present.

In our work, two genes, *cox1* and *nad1*, were used simultaneously, which increased the efficiency of results in the diagnosis of genotypes. In a study performed by Šoba et al. [31] in Slovenia on 18 samples of patients with CE, a molecular method was used on *cox1*, *nad1*, *nad5*, and *rRNA* genes. Their results showed that the majority of genotypes belonged to G1 genotypes, which was consistent with the strain identified in the present study. In a joint study conducted in the northwest of Iran and eastern of Turkey using *cox1* and *nad1* on 90 animal samples, it has been shown that all Iranian samples and the majority of Turkish samples have G1 strain. The study has also uncovered that only one sample has G3 strain senso stricto [32]. In another study conducted in the north of Iran by Pezeshki et al. [33], sheep strain was the most prevalent strain in humans, goats, sheep, and cattle, which supports the results of our study. In two similar studies conducted by Parsa et al. [13, 34] in the center of Iran, the predominant strain in this region was G1 or sheep strain, the same as our finding.

In Iran, seven genotypes (G1–G7) have been isolated from animals and humans [28]. Herein, only G1 genotype was isolated from the collected FFPE samples, and it seems that sheep genotype G1 has a significant role in the transmission of *E. granulosus* s.s. to human in the northwest regions of the country [28]. However, in some areas, such as the center and east of the country and even North African countries, the G6 was the most prevalent genotype in sheep, cattle, camels, and humans [5, 35]. An earlier study has reported the presence of two genotypes, that is, G1 and G6, in nine human CE cases in the East of Iran; hence, the G6 was represented as the most frequent genotype in this part of the country [17]. In our study, the G6 strain was not found, possibly due to the presence of mountains in the northwest of Iran and the lack of camel herds.

Researchers often utilize more than one marker to enhance the result accuracy and better differentiate identified genotypes, as well as study the sequence diversity of *Echinococcus* isolates [36–39]. In the current study, the genetic markers of *nad1* were used together with *cox1*. Our data in this study, similar to other investigations, reflected that the results of the two studied genetic markers, *cox1* and *nad1* genes, were the same, though the differentiation power of the *cox1* marker was higher than the *nad1* marker. In a similar study conducted by Yousefi et al. [40] on 80 paraffin‐embedded human CE collected from the southwest of Iran, G1 was the only genotype detected in their study among all samples, which affirms the results achieved in the current study [40]. For genotyping *E. granulosus s.l*, most of the researchers use one or more genetic markers, concurrently. ITS1, *cox1*, *nad1*, *nad5* or *12S rRNA* are most commonly used markers [41] . Our analysis did not show any significant genetic diversity among the G1 genotype isolates, *cox1 nad1* genes. In the study of Jafari et al. [30], using *cox1* and *nad1* genes, 12 haplotypes were obtained from the G1 strain. They emphasized that *cox1* gene, due to having more nucleotide indices, has a special superiority over *nad1* gene in differentiating genotypes and detecting intraspecific differences. Results from our study represented that the G1 strain has a significant homology, compared to the reference genotypes, in the GenBank. The results of gene sequencing also confirmed the sheep genotype in the human FFPE samples, which is in line with previous findings performed on animal and human samples [17, 30]. The lower sequencing success rate despite successful PCR amplification may be related to DNA fragmentation and chemical modifications caused by formalin fixation and long‐term paraffin preservation of FFPE tissues. However, in the current study and many other recent studies in Iran [36–39], G1 was the most common *E. granulosus* genotype, and since this genotype is transmitted among dogs and sheep in Iran, it is assumed that *E. granulosus* transmission in the northwest of Iran occurs in this cycle and sheep‐dog control program could be very effective, if planned. Since the G1 genotype is mainly maintained in the sheep‐dog transmission cycle, identification of this genotype could help target control measures toward dog deworming, livestock vaccination, meat inspection, and prevention of infected offal consumption by dogs.

In this study, as the frequency of infected people living in rural areas was higher than urban residents, the risk assessment of infection in rural areas can be a prerequisite for control measures. Thus, in disease control planning, full control should be exercised over the extermination of infected animals in slaughterhouses, as well as over the strict prevention of illegal livestock slaughter. Appropriate health education for housewives, especially women living in rural areas, education for those who have contact with dogs could be significantly effective strategies for controlling the disease. Vaccines are available for livestock and usually administered yearly to sustain effective control transmission. Collecting stray dogs and regular deworming of domestic dogs are recommended. In addition to other control measures such as health education, proper disposal of slaughter offal, strict meat inspection [35].

There are some limitations in our study. The tissue samples of FFPE are a precious source of retrospective studies throughout the world. However, extraction of DNA from these samples is not as simple as extraction from fresh or alcohol preserved materials, due to the inhibitory effects of formalin on PCR. Despite various commercial specialized kits are available for extracting DNA from FFPE tissues, many isolates do not shown valid results when the PCR protocols are used. For this reason, researchers who work in countries with endemic diseases such as CE prefer to utilize a fresh germinal/protoscolece layer of human hydatid cyst instead of using the FFPE or alcohol preserved isolates. Therefore, limited studies of *E. granulosus* have been investigated using FFPE tissues as the DNA source.

## 5. Conclusion

In conclusion, although similar studies have been performed in other parts of Iran, the present research was conducted in a unique tri‐border region adjacent to Turkey, Iraq, and the Nakhchivan Autonomous Republic of Azerbaijan. This location is characterized by intense cross‐border movement of people, livestock, and goods, which may facilitate transboundary transmission of *E.granulosus*. These epidemiological conditions distinguish our findings from previous reports and highlight their value for both national and regional control programs.

Along with effective vaccines for dogs, genotyping is a useful tool for prevention and control of CE and improve diagnostic methods for disease and efficient treatments and statistical mechanisms to better assess treatment costs.

## Author Contributions

Shahram Khademvatan and Saber Gholizadeh: study design, study execution, and interpretation, and preparation of the manuscript. Mahsa Boustani and Farahnaz Noroozinia: data collection, study execution, data analysis, and preparation of the manuscript.

Elham Yousefi: study execution and preparation of the manuscript.

## Funding

This study was supportd by the Urmia University of Medical Sciences, 10.13039/501100016286, IR.UMSU.REC.1398.242.

## Ethics Statement

This study was conducted according to established ethical guidelines, and informed consent obtained from the participants and approved by Research Ethics Committees of Urmia University of Medical Sciences (no: IR.UMSU.REC.1398.242, 2019‐10‐02).

## Conflicts of Interest

The authors declare no conflicts of interest.

## Data Availability

The data that support the findings of this study are available from the corresponding author upon reasonable request.
